# The Pyrland Multidisciplinary Teaching Project: Reflections on developing a teaching programme to improve the physical care of patients in an older adult inpatient setting

**DOI:** 10.1192/j.eurpsy.2026.11961

**Published:** 2026-07-31

**Authors:** B. Wills, J. Williams, J. Bruce, L. Mlambo, G. Harry, W. Baker, S. De Souza

**Affiliations:** Old Age Inpatient Psychiatry, Somerset Foundation Trust, Taunton, United Kingdom

## Abstract

**Introduction:**

Our psychiatric unit, like many others across Europe, is on a site away from the main hospital in town. We also receive patients from across the county. Therefore, when a patient is admitted to our ward, the doctors and nurses are responsible for their physical as well as psychiatric healthcare needs. Managing physical health care needs and medical emergencies on an inpatient psychiatric ward can pose significant challenges. We want to ensure that all ward staff feel confident in recognising and responding appropriately to acute physical healthcare conditions that may arise on an old-age inpatient psychiatric ward.

**Objectives:**

To design a programme to improve the physical healthcare of patients on an inpatient psychiatric ward through staff education, focussing on a multidisciplinary team (MDT) approach. To incorporate simulation sessions in the teaching programme, allowing for learning to be put into practice and to further team cohesion. To use MDT feedback to tailor the programme’s content and delivery.

**Methods:**

Through discussion with key stakeholders, we developed a curriculum of training focused on the most common medical emergencies and presentations seen on the ward. Topics include hypo/hyperglycaemia, chest pain, falls, seizures, pain control and the deteriorating patient in the community setting. Teaching sessions were carefully tailored to benefit all roles that may be involved in these scenarios. We also worked with our simulation fellow to employ in situ simulation of taught topics. For example, the assessment and management of a patient having a seizure or who has fallen.

**Results:**

Following liaison with the nursing team, we developed a series of didactic lecture and simulation sessions. These covered physical health care and management of medical emergencies and acute situations in the mental health setting. The programme was designed for weekly sessions on the ward, delivered by different members of the MDT. Qualitative feedback forms were developed for distribution following sessions to ensure the content is clear and relevant for all MDT members.

**Conclusions:**

We believe that the teaching sessions will equip the multidisciplinary team to better manage our patient’s health and wellbeing. We believe they will also increase confidence within the multidisciplinary team to recognise the unwell patient, in turn enabling timely and appropriate medical management. Once successfully implemented on our wards, we aim to roll this teaching programme throughout other inpatient psychiatry wards to help improve the physical healthcare management of more patients.

**Disclosure of Interest:**

None Declared

